# Microstructural Degradation and Creep Property Damage of a Second-Generation Single Crystal Superalloy Caused by High Temperature Overheating

**DOI:** 10.3390/ma16041682

**Published:** 2023-02-17

**Authors:** Xiaotong Guo, Hao He, Fangzhou Chen, Jiahao Liu, Wendao Li, Hao Zhao

**Affiliations:** 1 Chongqing CEPREI Industrial Technology Research Institute Co., Ltd., Chongqing 401332, China; 2 China Electronic Product Reliability and Environmental Testing Research Institute, Guangzhou 511370, China; 3 School of Materials Science and Engineering, Xiangtan University, Xiangtan 411105, China

**Keywords:** superalloy, overheating, creep property, microstructure

## Abstract

Nickel base superalloys are widely used to manufacture turbine blades, and overheating poses a serious threat to the safe service of turbine blades. In this study, a second-generation nickel base single crystal superalloy was taken as the research object, and we carried out the overheating treatment at 1100 °C and 1300 °C, and then tested the creep properties at 1000 °C/300 MPa and 1100 °C/130 MPa. Through systematic analysis of creep properties, γ/γ’ phases, and creep voids, the effects of overheating on the microstructures and creep properties of the experimental superalloy were revealed. The results demonstrate that the effect of overheating at 1100 °C on the microstructure of the experimental superalloy can be ignored, and the effect on the creep property is limited. The degree of γ’ dissolution is gradually increased and the creep property is reduced with overheating time extending at the overheating temperature of 1300 °C.

## 1. Introduction

Nickel base single crystal superalloys are more and more widely used to manufacture turbine blades and other core components of aeroengines due to their excellent comprehensive properties [1]. However, turbine blades may be subjected to overheating during service, resulting in severe degradation of the microstructure and mechanical properties. Rowe and Weiss et al. [2,3] found as early as the 1950s and 1970s that overheating would affect the mechanical properties of superalloys, and the effects on superalloys with different compositions were significantly different. For example, after overheating treatment at 982 °C, the creep property of IN700 alloy is not affected, but the creep property of M252 alloy is improved [4]. However, the correlation between microstructure and mechanical properties has not been fully established. Until the beginning of the 21st century, Cormier et al. [5,6,7] began to carry out research on nickel base single crystal superalloys, which found that the γ’ phase degraded obviously and the creep property decreased significantly due to overheating.

The studies on overheating in China have gradually received attention, and studies on overheating effect on forged superalloys [8], equiaxed casted superalloys [9,10], and directionally solidified superalloys [11,12] have been carried out. The results show that overheating, especially the thermal cycling condition [13], has a significant impact on the creep properties of superalloys. The creep rate of the alloy increases obviously at the overheating stage, resulting in a significant decrease in the creep life [11,14]. However, the public reports on the degradation of single crystal superalloys in overheating service are still limited in the domestic.

At present, the second-generation single crystal superalloy is still the mainstream material used in advanced aeroengines. Therefore, this study takes a second-generation domestic single crystal superalloy as the research object to investigate the microstructure degradation of the alloy after 5–120 min overheating at 1100–1300 °C and its effect on creep properties.

## 2. Materials and Methods

The experimental alloy is a second-generation nickel base single crystal superalloy, which has the advantages of excellent high temperature strength, casting processing properties, and stable microstructures. Compared with the first-generation nickel base single crystal superalloy, its high temperature bearing capacity is increased by about 40 °C. The experimental superalloy is suitable for manufacturing high temperature components such as turbine blades with complex cavities working below 1100 °C. The chemical composition of the experimental alloy is Ni-9.0Co-9.0W-7.5Ta-5.6Al-4.3Cr-2.0Mo-2.0Re-0.5Nb-0.1Hf-0.006C.

The experimental superalloy was produced by a high gradient vacuum directional solidification furnace (HRS) using the spiral crystal selection method. The test bars are 170 mm long and 12 mm in diameter. The crystal orientation of the bars is determined by the X-ray method. The single crystal test bars with [001] orientation within 8° of the principal stress axis were selected. The standard heat treatment of the experimental alloy is a solution heat treatment (1290 °C/1 h+1300 °C/2 h+1315 °C/4 h with air cooling (AC)) and an aging heat treatment (1120 °C/4 h, AC; 870 °C/32 h with AC). All the experimental solid bars had undergone the standard heat treatment in this study.

**Figure 1:**
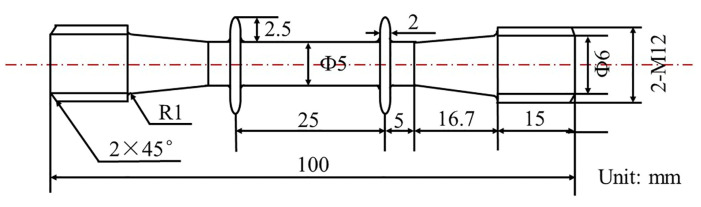
Sketch of the creep specimens.

To study the effect of overheating on the microstructure evolution, the overheating temperature was set at 1100 °C and 1300 °C, and the overheating duration was set as 5 min, 60 min, and 120 min, followed by air cooling. The solid bars after overheating treatment are sectioned into creep specimens. The sketch of the creep specimens is shown in Figure 1, which had a diameter of 5 mm and a length of 25 mm within the gauge length. The creep tests were carried out at 1000 °C/300 MPa and 1100 °C/130 MPa in air according to national standard GB/T 2039-2012, which is similar to International Standard ISO 204: 2009. An RDL-100 creep machine produced by Sinotest Equipment Co., Ltd. equipped with HEIDENHAIN ST 1288 extensometer was used to conduct the creep tests. The heating rate was set as 300 °C/h, and the stress was kept 200 N during the heating process. The creep stress started loading after the specimens kept at the creep temperature for 0.5 h, and the stress kept constant during the whole creep process. For the overheated and creep specimens, the fracture morphologies and microstructures were observed using a NIKON LV150 optical microscope (OM), ZEISS SUPRA 55 and a FEI Inspect F50 field-emission scanning electron microscope (FE-SEM). The average size of the γ’ precipitates was measured using Image-pro software, and the volume fraction of the γ’ precipitates was determined using the posture method and PS software.

## 3. Results and Discussions

### 3.1. Microstructure Characterization after Overheating

**Figure 2:**
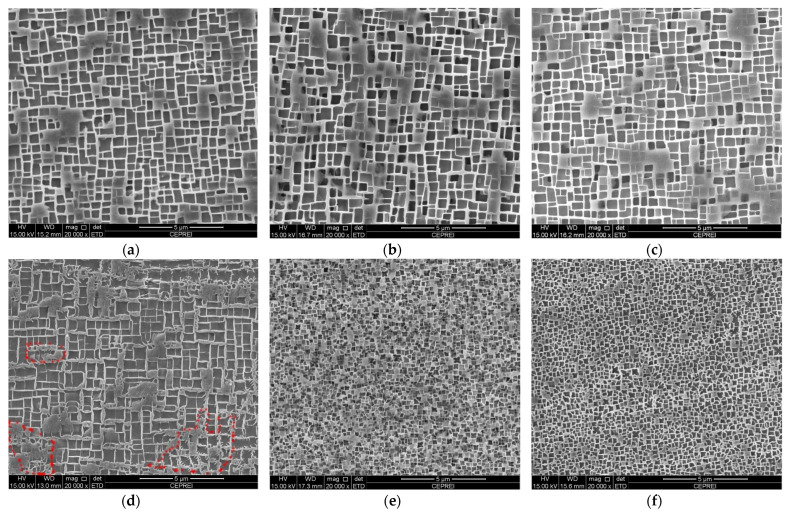
SEM-SE images of the γ/γ’ phases in the dendrite core regions of the experimental superalloy after overheating at (**a**) 1100 °C/5 min; (**b**) 1100 °C/60 min; (**c**) 1100 °C/120 min; (**d**) 1300 °C/5 min; (**e**) 1300 °C/60 min; (**f**) 1300 °C/120 min.

Figure 2 presents SEM-SE images showing the morphologies of γ/γ’ phases in the dendrite core regions of the investigated superalloy after overheating at 1100 °C and 1300 °C. The γ’ precipitates maintained a relatively cuboidal morphology after overheating at 1100 °C (Figure 2a–c), which was close to that after the standard heat treatment, indicating that the γ’ precipitates were basically not degraded. The γ’ precipitates were obviously dissolved when overheating temperature increased to 1300 °C, and the overheating duration had an obvious effect on the dissolution degrees. Figure 2d shows the morphology of γ’ precipitates after overheating at 1300 °C for 5 min. The γ’ precipitates were partially dissolved or coarsened, making the edges and corners uneven and serrated, as shown in the red box. When the overheating duration was extended to 60 min or 120 min, the size of the γ’ precipitates was obviously smaller and evenly distributed, indicating that the primary γ’ phase had been completely dissolved after the overheating treatment. The fine and uniform γ’ particles were secondary γ’ precipitates forming during the cooling process after the overheating treatment (see Figure 2c,d).

The volume fraction and average size of the γ’ precipitates in the dendrite core regions were counted after overheating, and the results are listed in Table 1. It is shown that the volume fraction and average size of the γ’ precipitates were 66.2% and 0.46 μm, 65.6% and 0.48 μm, 64.4% and 0.50 μm after overheating at 1100 °C for 5 min, 60 min, and 120 min, respectively. With the extension of the overheating duration, the volume fraction and average size of the γ’ precipitates decreased slightly, but was close to that after the standard heat treatment [15]. When the overheating temperature reached 1300 °C, the volume fraction of the γ’ precipitates was reduced to about 60%, and the average size was even reduced to 0.24 μm. For example, after overheating at 1300 °C for 60 min, the volume fraction and average size of the γ’ precipitates were reduced to 58.4% and 0.24 μm, respectively. Obviously, the laws presented by the statistical data results of the γ’ phase were consistent with those presented in Figure 2.

**Figure 3:**
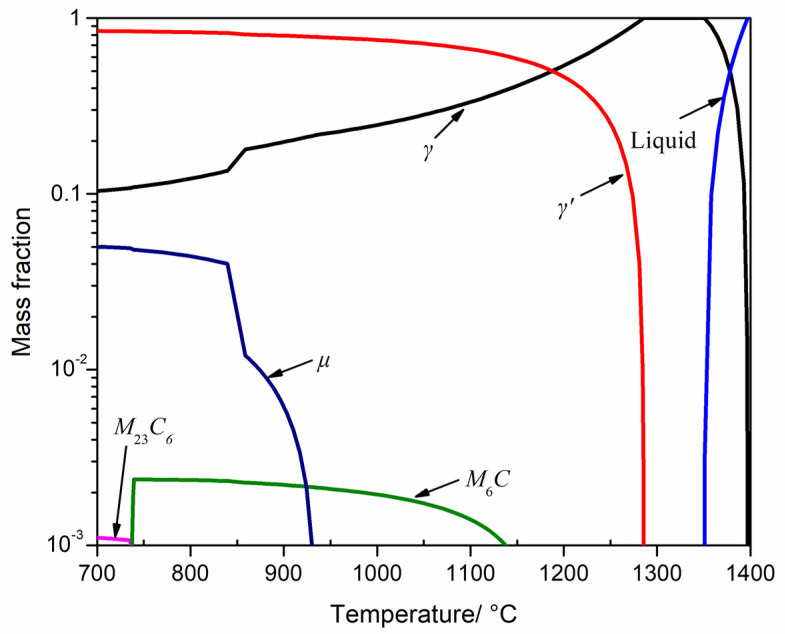
Phase equilibrium diagram of the experimental superalloy between 700 °C and 1400 °C using Pandat software 2018 version based on PanNi 2018 database.

Figure 3 shows the phase equilibrium diagram of the investigated alloy between 700 °C and 1400 °C, the relevant data are listed in Table 2. Figure 3 further demonstrates that the equilibrium mass fraction of the γ’ phase is 66.7% and 0 at 1100 °C and 1300 °C, respectively. Therefore, when the experimental superalloy was exposed to overheating at 1100 °C, no obvious degradation of the γ’ phase occurred (Figure 2a–c). The γ’ phase dissolution temperature is much higher than 1100 °C, but lower than 1300 °C (Figure 3). Through analysis of the slope change of the mass fraction vs. temperature curve, it can be figured out that when the temperature rises from 700 °C, the γ’ phase content decreased slowly. When the temperature rises to about 1200 °C, the γ’ phase content began to decrease significantly. At 1300 °C, the primary γ’ precipitates trend to completely dissolve back into the γ matrix. The above thermodynamic calculation results are consistent with the experimental results (Figure 2 and Figure 3).

Table 2 lists the mass fraction of γ phase, M_6_C carbides, M_23_C_6_ carbides, and μ phase at the equilibrium state of 1100 °C and 1300 °C. Only the mass fraction of M_6_C carbide is larger than 0, while also small enough to be ignored. Because no M_6_C carbides, M_23_C_6_ carbides, or μ phase precipitates were found in the standard heat-treated microstructure, and there was no precipitation power for the three precipitates at the overheating temperatures, thus no precipitation behavior of the M_6_C carbides, M_23_C_6_ carbides, and μ phase was found in the overheated microstructure.

### 3.2. Creep Properties after Overheating

**Figure 4:**
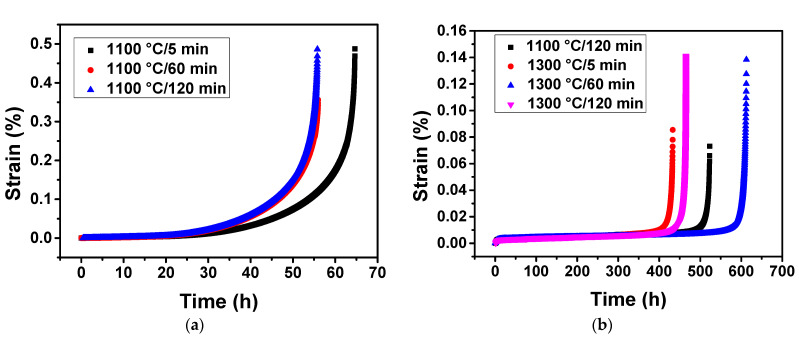
Curves of creep strain vs. time under creep condition 1000 °C/300 MPa for specimens overheated at 1100 °C/5 min, 1100 °C/60 min, and 1100 °C/120 min (**a**); under creep condition 1100 °C/130 MPa for specimens overheated at 1100 °C/120 min, 1300 °C/5 min, 1300 °C/60 min, and 1300 °C/120 min (**b**).

Figure 4a illustrates the creep strain vs. time curve under 1000 °C/300 MPa of the experimental superalloy after overheating at 1100 °C. The results show that the superalloy had the longest creep life of 64.6 h after being exposed to 1100 °C/5 min. The creep life of the superalloy was 55.9 h and 55.7 h after being exposed to 1100 °C/60 min and 1100 °C/120 min, respectively. This means that the creep life of the superalloy was obviously reduced when the overheating duration was extended from 5 min to 60 min. However, when the overheating duration was extended to 120 min, the creep life did not decrease significantly.

The γ’ phase is the main strengthening phase in nickel base superalloys, and its volume fraction and size have a great influence on creep life [16]. Combined with the volume fraction and size statistics of the γ’ phase after overheating at 1100 °C (Table 1), a higher volume fraction and smaller average size of the γ’-phase precipitates may promote a longer creep life of the overheated specimens.

Figure 4b demonstrates the creep strain vs. time curve of the superalloy under 1100 °C/130 MPa after overheating at 1300 °C. The creep life of the superalloy was 523.2 h and 465.1 h after overheating exposure to 1100 °C/120 min and 1300 °C/120 min, respectively. The creep life of the superalloy was significantly reduced with the increase of overheating duration. On the other hand, the creep life of the superalloy was 433.2 h, 612.5 h, and 465.1 h after overheating for 5 min, 60 min, and 120 min, respectively. This indicated that the creep life of the superalloy was significantly increased when the overheating duration extended from 5 min to 60 min at 1300 °C, while the creep life of the alloy was significantly reduced when the overheating duration was extended to 120 min. The creep life was the longest under the measured conditions at 1300 °C/60 min, and the reasons need further analysis.

By comparing the volume fraction and size data of the γ’ phases after overheating (Table 1), we can confirm that overheating exposure to 1300 °C causes significant degradation of γ’ phases, and this is the main reason for the decrease of creep life. In general, the optimum volume fraction of γ’ phases in nickel base single superalloys is about 65% [17]. Considering the changes in average size were obvious, while the volume fraction of the γ’ precipitates was slightly lower than 65%, the significant decrease of the creep life was mainly due to the decreased size or the decreased volume fraction (Table 1, Figure 4b). In general, the creep life of nickel base superalloys reaches the maximum value at a certain size of γ’ precipitates, and reduces with a larger or smaller size [17]. In addition, the optimum size of γ’ phases may vary due to the change in the alloy composition. This study shows that for the experimental superalloy, the average size of the γ’ phases decreased from 0.50 μm to 0.24–0.29 μm would significantly reduce the creep life at 1100 °C/130 MPa.

**Figure 5:**
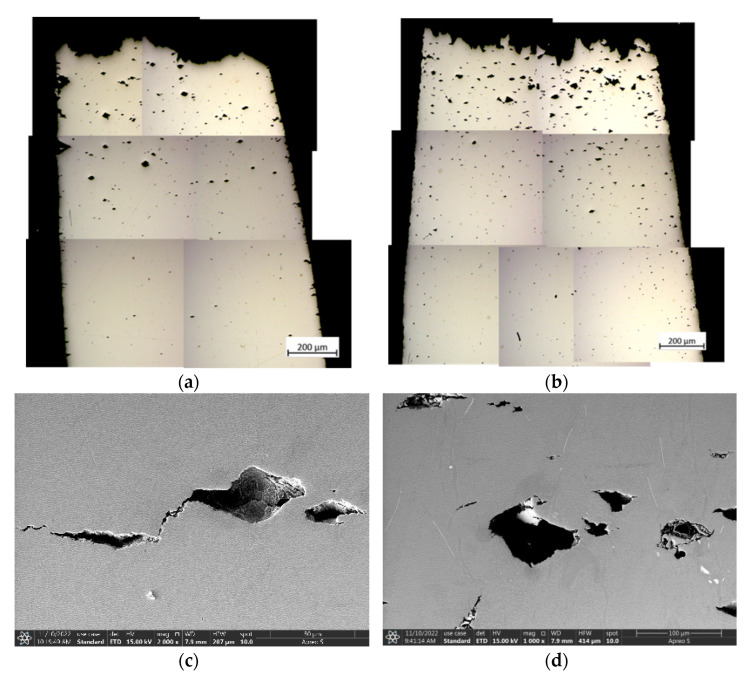
OM images of the longitudinal sections in the experimental superalloy after creep fracture at 1000 °C/300 MPa after overheating at 1100 °C/120 min (**a**) and 1300 °C/120 min (**b**); SEM images of the cracks in the longitudinal sections of the experimental superalloy after creep fracture at 1000 °C/300 MPa after overheated at 1100 °C/120 min (**c**) and 1300 °C/120 min (**d**).

The fracture morphology in the longitudinal section of the creep specimens under 1000 °C/300 MPa was observed. The results showed that there was a certain degree of necking near the fracture surface of each specimen. In addition, a large number of secondary cracks can be observed near the main cracks at the edge of the specimens. With the increase of the distance away from the fracture surface, the density and size of cracks and cavities in the specimen show a decreasing trend (Figure 5a).

By magnifying the creep cavities near the fracture surface of the specimens overheating exposed to 1100 °C, no TCP (topologically close-packed) phases were found around the cavities, as shown in Figure 5c. This shows that the voids existing in the experimental superalloy itself or generated in the creep process are primary factors for crack initiation and propagation, which is consistent with previous research experience [10].

Generally, the creep cracks of single crystal superalloys are mainly initiated on the pores formed in the existing loosening or creep process. Some studies believe that the formation process of regular micropores in creep specimens is related to dislocation movement [18]. The existence of as-cast micropores and solid solution micropores distributed among dendrites provides favorable nucleation sites for the formation of these creep micropores, which leads to more creep micropores in the interdendritic region than in the dendrite core region. At the third stage of creep, a large number of dislocations cut into the raft γ’ phase making the creep resistance reduced, the alternate slip makes γ’ raft twist and produce micro-holes or microcracks [19]. Then, the cavities aggregate, grow up and microcracks propagate in the progress of creep, leading to the increase of effective stress and deformation rate of creep, and the final fracture.

The metallographic microstructures in the longitudinal sections of the creep fracture specimens under 1100 °C/130 MPa were also investigated. The results showed that the necking phenomenon and cracks distribution law in the specimens at 1100 °C/130 MPa were basically consistent with those in the fracture specimens at 1000 °C/300 MPa. However, the size of creep cavities in the creep fracture specimens at 1100 °C/130 MPa was larger overall in comparison with that in the specimens at 1100 °C/300 MPa. In particular, the specimens subjected to overheating treatment at 1300 °C/120 min and then creep fracture at 1000 °C/300 MPa had the densest creep pores and cracks. This may be because more initial cavities and microcracks were produced in the experimental superalloy under the condition of overheating treatment at 1300 °C/120 min (Figure 5b). During the creep process, denser cavities are more prone to grow into creep cavities and serve as stress concentration points for crack initiation. This also reflects that the specimens after overheating treatment at 1300 °C/120 min had more serious microstructure degradation than the other specimens.

The creep holes and microcracks in the necking area of the creep fracture specimens at 1100 °C/300 MPa were magnified using the scanning electron microscope. The results showed that a certain amount of TCP phases precipitated in the necking zone of the creep fracture specimens. The TCP phases precipitated during the creep process at 1100 °C/130 MPa. This means that the creep temperature is the key factor leading to the precipitation of the TCP phases. Generally, the TCP phase is hard and brittle in superalloys. In addition, the TCP phases in the experimental alloy was needle shaped or sheet shaped (Figure 5d) and was easy to produce a large stress concentration during the load bearing process, leading to excessive local stress and a weak link in creep fracture. Therefore, for the creep specimens under the condition of 1100 °C/130 MPa, the possible important crack source was not only the holes in the superalloy itself or generated in the creep process, but also the acicular TCP phases precipitated in the γ matrix during the creep process. According to the equilibrium phase diagram (Figure 3), the TCP phase is an unstable phase and most likely μ phase, but the detailed structural characterization is still required.

**Figure 6:**
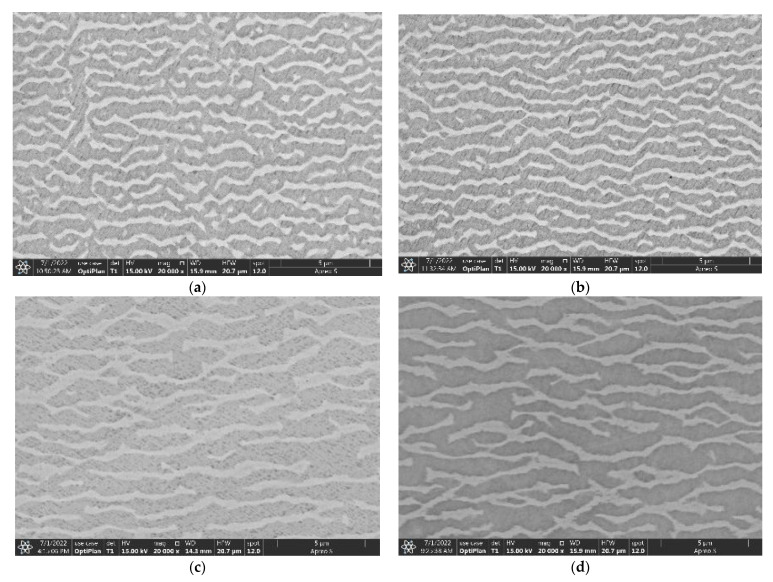
SEM-SE images of the γ’ precipitates in the dendrite cores of the longitudinal sections of the experimental alloy after creep fracture at 1000 °C/300 MPa after overheating exposed 1100 °C/5 min (**a**) and 1100 °C/120 min (**b**); SEM-SE images of the γ’ precipitates in the dendrite cores of the longitudinal sections of the experimental alloy after creep fracture at 1100 °C/130 MPa after overheating exposed 1100 °C/5 min (**c**) and 1100 °C/120 min (**d**).

Figure 6a,b present SEM-SE images showing the morphologies of γ’ precipitates in the dendrite cores of the longitudinal sections after creep fracture at 1000 °C/300 MPa after overheating exposed 1100 °C. The γ’ precipitates suffered obvious rafting (i.e., parallel to (001) plane) in the direction perpendicular to the stress, and a certain degree of coarsening connection occurred in the direction parallel to the stress. For the three kinds of specimens with overheating for 5 min, 60 min, and 120 min, the morphologies of the γ’ phase were basically close.

The morphologies of the γ’ precipitates in the dendrite core regions of the longitudinal sections after creep fracture at 1100 °C/130 MPa were also observed, and are shown in Figure 6c,d. The γ’ rafting also occurred in the fracture specimens, and the thickness of the γ’ rafts increased obviously in comparison with those of the fractured specimens at 1000 °C/300 MPa. On the one hand, this may be due to the fact that the creep temperature at 1100 °C was relatively high and was easy to cause γ’ raft. On the other hand, the volume fraction of the γ’-phases after overheating at 1100 °C was lower than that at 1000 °C, and was easy to form a thicker γ’ raft.

Studies have shown that the rafting of γ’ phase is considered to be a kind of microstructure evolution caused by the direction diffusion of elements due to the stress gradient generated by the superposition of γ/γ’ phases mismatch stress and external stress [20,21]. Thus, the rafting direction is determined by the external stress and γ/γ’ mismatch. The mismatch degree of the γ/γ’ phases is negative of the experimental alloy, so the “N” type raft perpendicular to the stress axis appeared under creep tensile stress (Figure 6c,d). The classical raft theory is based on the high-temperature creep behavior of nickel base superalloy, and can be summarized as follows [16]: (1) raft microstructure starts to form at the early stage of creep; (2) the increase of external stress promotes the rapid formation of raft microstructure; (3) once the macro strain exceeds the “threshold strain value”, raft microstructure starts to form. During the creep process at 1100 °C/130 MPa, the rafting phenomenon can only occur when the creep strain accumulates to a certain extent, that is, it breaks through a certain threshold strain value.

The microstructure characteristics of γ/γ’ phases have an important effect on the creep resistance of the superalloys. It is shown that the Orowan resistance needed to be overcome when the dislocation bypasses the γ’ phase is closely related to width of γ channel. The larger the γ channel width, the smaller the Orowan resistance, and the lower the creep resistance of the alloy. For the experimental alloy in this study, with the increasing of γ channel width and the decreasing of Orowan resistance, the creep life of the alloy also showed a decreasing trend (Figure 6). It can be inferred that the smaller the thickness of the γ’ phase, the more interfaces of γ/γ’ two phases. The dislocation network formed at the γ/γ’ interfaces plays an important role in hindering the further movement of dislocations. With the thickness of γ’ phase in the experimental superalloy decreasing, the creep resistance may increase because of more dislocations met the γ/γ’ phase interfaces.

The parameter Ω of rafting is used to characterize the integrity of raft microstructure, and its definition formula is:

(1) $$\Omega =P_{L}^{⊥}{-P}_{L}^{//}$$

wherein P_L_ represents the number of crosses and interruption of raft microstructure within the unit length in a specified direction (i.e., the number of the γ/γ’ phase interfaces). “⊥” and “//” represent the number of *P*_L_ along the vertical and parallel raft directions, respectively. The value range of Ω is 0–1. When the value of Ω is 0, it represents the equiaxed γ’ phase microstructure, that is, vertical and parallel are equivalent directions. When the value of Ω is 1, it represents the ideal integrity γ’ phase raft microstructure, that is the phase γ’ raft is neither interrupted nor bifurcated. The closer the value of Ω to 1, the more perfect the γ’ phase raft structure [22].

**Figure 7:**
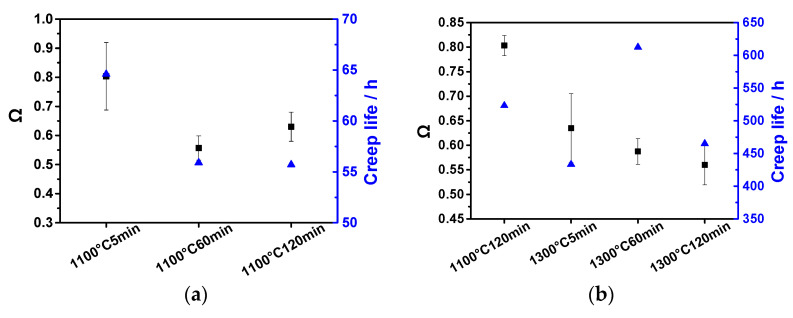
The integrity of γ’ raft (Ω) in the dendrite core regions of the longitudinal sections of the experimental alloy and creep life under 1000 °C/300 MPa (**a**) and 1100 °C/130 MPa (**b**) after suffering overheating treatments.

The integrity of the γ’ raft is statistically analyzed and compared with the creep life. The results showed that there was a certain difference in the integrity of γ’ rafting formed in the experimental superalloy under the creep condition of 1000 °C/300 MPa (Figure 7a). The specific performance was that the specimens overheated treated at 1100 °C/5 min had more complete rafting structures, while the integrity of the γ’ rafting structures of the two specimens with longer overheating duration decreased significantly. The creep life was closely related to γ’ raft integrity, and the specimens with higher raft integrity had a longer creep life.

It has been shown that the single crystal superalloy with a straight and complete raft structure during creep can obtain better creep properties than that with bent and bifurcated raft structures [23]. The higher the integrity of raft microstructure (i.e., the closer the value of Ω was to 1), the more γ/γ’ phase interfaces, the fewer the γ phase channels, the more the movement of dislocations parallel to the stress axis is blocked, the slower creep rate. Therefore, for the specimen overheating treated to 1100 °C/5 min, the complete raft structure (with the highest Ω value) had made a certain contribution to its excellent creep performance (Figure 4a).

The integrity of γ’ raft in the creep specimens on the condition of 1100 °C/130 MPa for different overheated specimens had a certain difference. The specimens overheating treated to 1100 °C/120 min had a complete raft structure, and the integrity of γ’ raft decreased significantly with the increase of the overheating duration. the creep life was closely related to the raft integrity, and the specimens with higher raft integrity had a longer creep life. This is because the higher raft integrity, the more γ/γ’ phase interfaces, the more the movement of dislocations parallel to the stress axis was blocked, and the creep rate decreased.

## 4. Conclusions

In summary, we researched the microstructural degradation and creep property damage of a second-generation nickel base single crystal superalloy after overheating. The experimental superalloy was exposed to overheating treatments at 1100 °C and 1300 °C with different overheating durations, and then tested the creep properties at 1000 °C/300 MPa and 1100 °C/130 MPa, respectively. The microstructures and creep properties of the experimental superalloy were comparatively studied. The results are summarized as follows:

(1) The γ’-phase was basically not degraded with maintaining a relatively cuboidal morphology after overheating at 1100 °C, indicating that the effect of overheating at 1100 °C on the microstructure of the experimental superalloy can be ignored However, the γ’-phase was obviously dissolved when overheating temperature increased to 1300 °C. The overheating duration had an obvious effect on the size of the γ’-phase, which was obviously smaller and evenly distributed. Meanwhile, there were no M_6_C carbides, M_23_C_6_ carbides, or μ phase precipitates in the γ matrix after overheating, because there is no precipitation power for the three precipitates at the overheating temperatures.

(2) For the experimental superalloy, the creep life was 64.6 h and 55.7 h after exposed to 1100 °C/5 min and 1100 °C/120 min, respectively, indicating that the creep life was significantly reduced with the increase of overheating duration. This is due to the fact that there are a higher volume fraction and smaller size of the γ’-phase in a shorter overheating time, thus can promote a longer creep life. Additionally, the creep life was 523.2 h and 465.1 h after overheating exposure to 1100 °C/120 min and 1300 °C/120 min, respectively. The creep life was significantly reduced with the increase of overheating temperature. This is because overheating exposure to 1300 °C cause significant degradation of the γ’ phases and more initial cavities and microcracks were produced.

(3) For the creep fracture specimens, the γ’ precipitates suffered obvious rafting (i.e., parallel to (001) plane) in the direction perpendicular to the stress, and a certain degree of coarsening connection occurred in the direction parallel to the stress. The creep life was closely related to the raft integrity, and the specimens with a higher raft integrity had a longer creep life. This is because the higher raft integrity, the more γ/γ’ phase interfaces, the more the movement of dislocations parallel to the stress axis was blocked, and the creep rate decreased.

## Figures and Tables

**Table 1:** The statistical volume fraction and average size of γ’ particles in the dendrite core regions of the experimental superalloy after overheating.

Overheating Condition	Volume Fraction/%	Average Size/μm
1100 °C/5 min	66.2 ± 1.2	0.46 ± 0.02
1100 °C/60 min	65.6 ± 1.4	0.48 ± 0.04
1100 °C/120 min	64.4 ± 1.2	0.50 ± 0.05
1300 °C/5 min	58.0 ± 2.3	0.29 ± 0.04
1300 °C/60 min	58.4 ± 1.6	0.24 ± 0.03
1300 °C/120 min	60.5 ± 2.5	0.24 ± 0.01

**Table 2:** Precipitation temperature range and mass fraction of the secondary phases in the experimental superalloy between 700 °C and 1400 °C based on Pandat software.

Phase	Precipitation Temperature/%	Mass Fraction at 1100 °C	Mass Fraction at 1300 °C
γ’	<1285.6	66.7	0
M_6_C	735.8–1189.6	0.1	0
M_23_C_6_	<738.9	0	0
μ	<935.8	0	0

## Data Availability

Not applicable.
